# Effect of wind farms on wintering ducks at an important wintering ground in China along the East Asian–Australasian Flyway

**DOI:** 10.1002/ece3.6701

**Published:** 2020-08-20

**Authors:** Shanshan Zhao, Huan Xu, Ningning Song, Zhenghuan Wang, Ben Li, Tianhou Wang

**Affiliations:** ^1^ School of Life Science East China Normal University Shanghai China; ^2^ Institute of Eco‐Chongming (IEC) Shanghai China

**Keywords:** Anatidae, coastal wetlands, East Asian–Australasian Flyway, landscape, wind energy development

## Abstract

Wind farms offer a cleaner alternative to fossil fuels and can mitigate their negative effects on climate change. However, wind farms may have negative impacts on birds. The East China Coast forms a key part of the East Asian–Australasian Flyway, and it is a crucial region for wind energy development in China. However, despite ducks being the dominant animal taxon along the East China Coast in winter and considered as particularly vulnerable to the effects of wind farms, the potential negative impacts of wind farms on duck populations remain unclear. We therefore assessed the effects of wind farms on duck abundance, distribution, and habitat use at Chongming Dongtan, which is a major wintering site for ducks along the East Asian–Australasian Flyway, using field surveys and satellite tracking. We conducted seven paired field surveys of ducks inside wind farm (IWF) and outside wind farm (OWF) sites in artificial brackish marsh, paddy fields, and aquaculture ponds. Duck abundance was significantly higher in OWF compared with IWF sites and significantly higher in artificial brackish marsh than in aquaculture ponds and paddy fields. Based on 1,918 high‐resolution satellite tracking records, the main habitat types of ducks during the day and at night were artificial brackish marsh and paddy fields, respectively. Furthermore, grid‐based analysis showed overlaps between ducks and wind farms, with greater overlap at night than during the day. According to resource selection functions, habitat use by wintering ducks was impacted by distance to water, land cover, human activity, and wind farm effects, and the variables predicted to have significant impacts on duck habitat use differed between day and night. Our study suggests that wintering ducks tend to avoid wind turbines at Chongming Dongtan, and landscape of paddy fields and artificial wetlands adjoining natural wetlands is crucial for wintering ducks.

## Graphical abstract

We assessed the effect of wind farm on duck abundance, distribution, and habitat use using field surveys and satellite tracking at Chongming Dongtan, China, along the East Asian–Australasian Flyway. Our results suggest that ducks tend to avoid wind turbines at Chongming Dongtan, and landscape of paddy fields and artificial wetlands adjoining natural wetlands is crucial for wintering ducks.

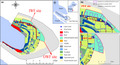

## 1 INTRODUCTION

Wind farms offer a cleaner alternative to fossil fuels and can mitigate their negative effects on climate change; however, they have several complex ecological consequences (Kuvlesky et al., 2007; Thaker, Zambre, & Bhosale, 2018), especially in terms of their potential negative effects on birds (Gómez‐Catasús, Garza, & Traba, 2018; Kunz et al., 2007; Reid, Krüger, Whitfield, & Amar, 2015). These effects fall into three categories: (a) direct mortality as a result of collision with wind turbines and their associated facilities (Aschwanden et al., 2018; Graff, Jenks, Stafford, Jensen, & Grovenburg, 2016; Plonczkier & Simms, 2012); (b) effects of wind farm‐related visual, electromagnetic radiation, high noise, and vibration disturbances on bird distribution (LeBeau et al., 2017; Winder, Gregory, McNew, & Sandercock, 2015); and (c) temporal and spatial habitat displacement due to habitat loss and/or disturbance, or by creating barriers to bird movements (Dohm, Jennelle, Garvin, & Drake, 2019; Gómez‐Catasús et al., 2018). Distribution changes and habitat displacement/changes caused by wind farms could have individual‐ or population‐level effects regionally and globally, including impacts on survival, breeding success, energy expenditure, and bird‐community structure and stability (Dahl, Bevanger, Nygård, Røskaft, & Stokke, 2012; Gómez‐Catasús et al., 2018; LeBeau et al., 2017; Marques et al., 2020). These effects may eventually lead to declines in bird species and abundance at different levels, and have thus received considerable attention (Dhunny, Allam, Lobine, & Lollchund, 2019; Marques et al., 2020; Thaxter et al., 2019).

China has become the largest developer of wind energy since 2008, and its wind power installations generated 21.2 GW in 2018 (Global Wind Energy Council, 2019). The East China Coast is one of the most important regions for wind energy developments, due to its high human population density, economic development, and electricity demand. However, it is also an important part of the East Asian–Australasian Flyway, which is one of the most important global waterbird flyways (Bamford, Watkins, Bancroft, Tischler, & Wahl, 2008; Cao, Zhang, Barter, & Lei, 2010), and includes the highest proportion (19%) of threatened waterbird populations in the world. The Yangtze River estuary, Jiangsu coasts, Yellow River estuary, and East Liaoning Bay in this area are important wintering grounds for ducks and cranes along the East Asian–Australasian Flyway (Bai et al., 2015; Barter & Wang, 1990; Choi, Battley, Potter, Rogers, & Ma, 2015). However, numerous wind farms have been installed or proposed in these areas (National Development & Reform Commission of the People’s Republic of China, 2016).

The East China Coast covers the coasts from Liaoning to Hainan provinces (~8,000 km) (Bai et al., 2015; Duan et al., 2020; Xia et al., 2017). Ducks comprise the dominant animal taxon along the East China Coast in winter (Cao, Barter, & Lei, 2008). As one of most important parts of the Yellow Sea, the intertidal mudflats of the East China Coast north of the Yangtze River mouth support duck populations dominated by dabbling ducks, particularly the Eastern spot‐billed duck *Anas zonorhyncha* (100,000 individuals) and the Mallard *Anas platyrhynchos* (110,000 individuals) (Bai et al., 2015; Cao et al., 2008). However, ducks are particularly vulnerable to wind farms (Meattey et al., 2019); they inhabit shallow, subtidal coastal regions (Bengtsson et al., 2014), which are also favored regions for onshore wind farm developments because of their abundant wind resources and low construction costs compared with offshore wind farms (He, Xu, Shen, Long, & Yang, 2016). Chongming Dongtan is located in the Yangtze River estuary, China, and serves as a wintering site for ducks and an important stopover site for shorebirds migrating between Australia and Siberia (Barter & Wang, 1990; Iwamura et al., 2013; Zou et al., 2016). Wind farm developments at Chongming Dongtan started in 2005, and more than 100 wind turbines have since been constructed. However, studies of the relationship between wind farms and ducks in this region are lacking, and the potential negative impacts of the wind farms on ducks remain unknown.

Many studies have focused on the impacts of offshore wind farms on ducks, especially in Europe (Desholm & Kahlert, 2005; Dierschke, Furness, & Garthe, 2016; Guillemette & Larsen, 2002; Plonczkier & Simms, 2012) and North America (Loring et al., 2014; Meattey et al., 2019). Studies of direct mortality of ducks caused by wind energy facilities showed that the collision risk was minimal, due to the birds’ avoidance behavior (Desholm & Kahlert, 2005; Masden et al., 2009; Wang, Wang, & Smith, 2015). However, wind farms were predicted to have indirect effects on populations, including changes in community composition (Dierschke et al., 2016), distribution patterns (Guillemette & Larsen, 2002), and disturbance and displacement (Bradbury et al., 2014; Kelsey, Felis, Czapanskiy, Pereksta, & Adams, 2018; Masden et al., 2009), which were considered to have a greater impact than the direct effects (Meattey et al., 2019). Hötker (2017) classified ducks as one of the most severely affected and displaced groups of species, and suggested that they might abandon suitable habitat within or close to a wind farm, or use it less frequently than they would in the absence of the wind farm. Recent studies indicated the environmental variables likely to influence duck abundance, distribution, and habitat use at offshore wind farms (Fernández‐Bellon, Wilson, Irwin, & O’Halloran, 2019; Larsen & Guillemette, 2007) and at onshore wind farms located inland (Loesch et al., 2013). Habitat type was especially important, with some studies suggesting that habitat type would have a stronger effect on ducks than the presence of wind farms (Hötker, 2017; Loesch et al., 2013). However, the impact of onshore wind farms along the East China Coast on ducks is less well known, and there is thus an urgent need to qualify the effects of habitat type and wind farms on ducks in relation to wind energy development in this region.

Tracking data based on radar, radio telemetry, and satellite tracking, rather than field surveys, have commonly been used to record duck movements, identify key habitats, and assess collision risks around offshore wind farms, mainly focusing on tracking individuals (Desholm & Kahlert, 2005; Dierschke et al., 2016; Loring et al., 2014; Meattey et al., 2019; Plonczkier & Simms, 2012). However, field surveys conducted during the day at community population level have been used to investigate duck abundance and flight behavior in response to onshore wind farms (Loesch et al., 2013). It is therefore necessary to use both approaches to assess the effect of wind farms in coastal wetlands on wintering duck abundance based on field surveys (population level), and on the distribution and habitat use by dominant duck species based on tracking data (individual level).

In this study, we investigated the effects of onshore wind farms on wintering ducks in coastal wetlands along the East Asian–Australasian Flyway from November 2018 to February 2020, using Chongming Dongtan as a sample study site. We investigated the differences in duck abundance between sites inside and outside the wind farms by conducting seven duck field surveys in the three main habitats (artificial brackish marsh, paddy fields, and aquaculture ponds). On the basis of the data, we then quantified the dominant duck distributions and their potential overlaps with wind turbines by attaching global positioning system–global system for mobile communication (GPS/GSM) transmitters to 12 ducks (six Eastern spot‐billed ducks and six Mallards). We also explored the effects of multiple factors, especially wind turbines, on habitat use by ducks using resource selection functions (RSFs). These results in terms of the duck abundance, distributions, and habitat use of dominant duck species around wind farms identified crucial areas and landscapes in which wintering ducks may be vulnerable to ecological impacts from onshore wind farms along the coast. This information can help to provide practical recommendations to guide the future development and management of coastal wind farms along the East Asian–Australasian Flyway.

## 2 MATERIALS AND METHODS

### 2.1 Study area

**Figure 1:**
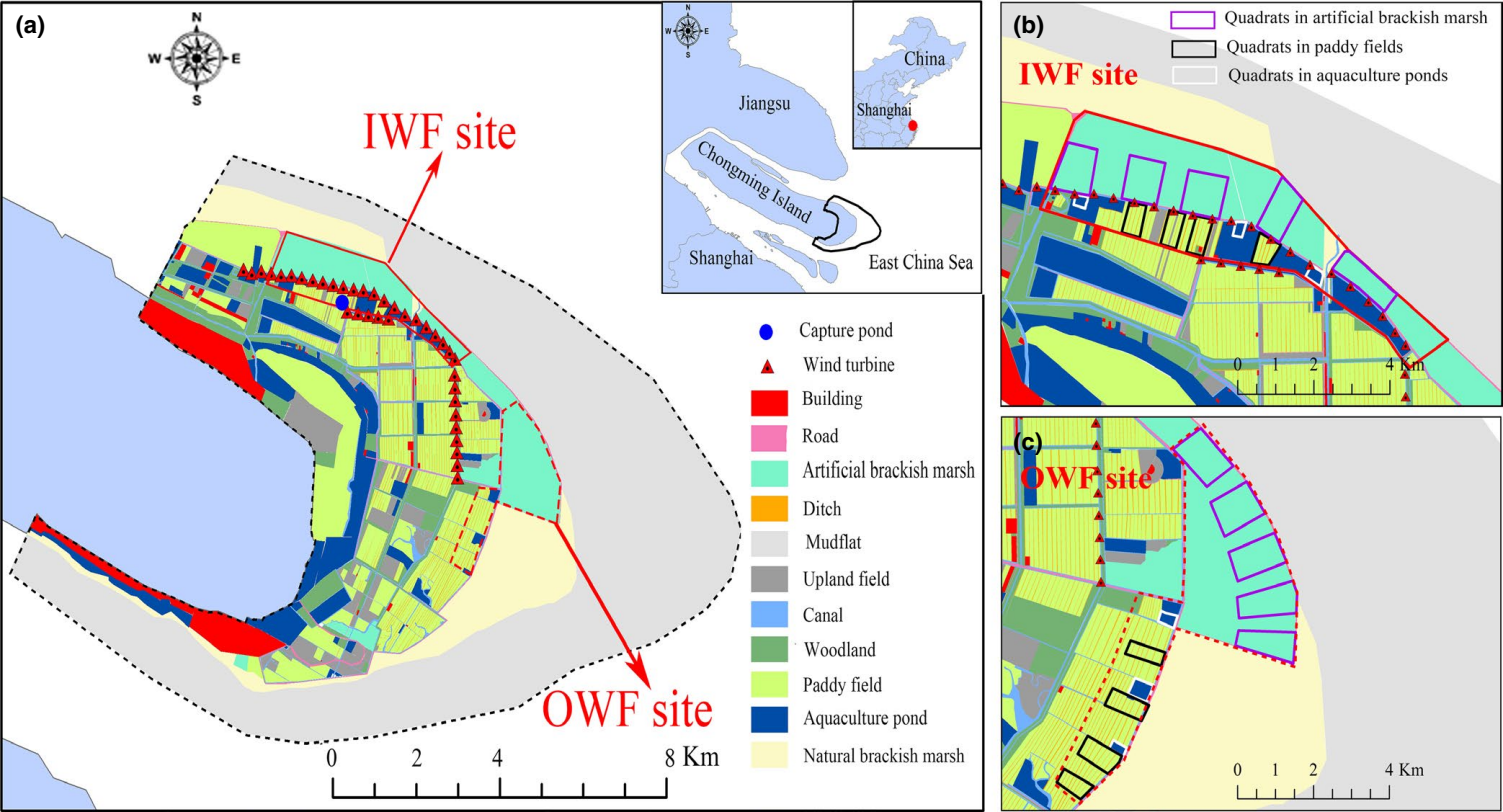
Landscape classification, wind turbine distributions (a), and duck survey quadrats at sites inside wind farms (IWF) (b) and outside wind farms (OWF) (c) at Chongming Dongtan, China

Chongming Dongtan (31°25′–31°38′N, 121°50′–122°05′E) is located at the eastern end of Chongming Island in the Yangtze River mouth, China (Choi et al., 2019) (Figure 1a). The main habitat types at Chongming Dongtan included mudflats, natural brackish marsh, artificial brackish marsh, farmland (mostly rice paddy fields), aquaculture ponds, and woodland (Zou et al., 2016). *Scirpus mariqueter, Spartina alterniflora*, and *Phragmites australis* were the main vegetations in the natural brackish marsh. Artificial brackish marsh, which formed a boundary between the natural marsh and intertidal mudflats, was a restoring wetland including sluice gates linking to the open water through canals in the natural marsh and mudflats, created by an engineering project aimed at removing the alien smooth cordgrass (*S. alterniflora*) and providing habitats for waterbirds (Kuang et al., 2019). A large area of paddy fields and aquaculture ponds was distributed inland of the dyke.

Chongming Dongtan is an important coastal site in terms of wind energy resources in Shanghai, and two wind farms (67.5 MW) started operating here between 2005 and 2012. The turbines were arranged linearly along the dyke between both brackish marshes and farmland/aquaculture ponds (Figure 1a). The height of the wind turbines in Chongming Dongtan was about 90 m, and the hub radius was about 45 m.

### 2.2 Duck surveys

Ducks overwintered at Chongming Dongtan from October to the following March (Ma et al., 2007, 2009), with stable species and abundance from November to the following February (Zou et al., 2016). We conducted seven surveys over two winters (four in November 2018 to February 2019, and three in November 2019 to January 2020). We classified the duck habitats into three main habitats, artificial brackish marsh, paddy fields, and aquaculture ponds, according to previous studies (Bengtsson et al., 2014; Ma et al., 2004; Niu, Zou, Yuan, Zhang, & Wang, 2013).

We assessed the potential effects of the wind farms using a paired survey design to quantify duck abundances at sites inside (IWF) and outside the wind farms (OWF) among the three habitats at Chongming Dongtan (Figure 1a). We defined the OWF site as at least 1.30 km from the wind turbines, which was regarded as the affective distance for ducks at Chongming Dongtan (Li et al., 2020). The microhabitats of the IWF and OWF sites were consistent. We classified the three habitat types at the IWF and OWF sites based on a total land cover of the respective habitat of >70%. We then selected quadrats in each habitat, with ≥0.50 km between quadrats to minimize both their effect on each other and the autocorrelation of differences between quadrats. The quadrat size depended on the land use, based on field surveys. Along the dyke at Chongming Dongtan, 10 quadrats were located in artificial brackish marsh (five IWF and five OWF) near the outside of the dyke, eight in paddy fields (four IWF and four OWF), and six in aquaculture ponds (three IWF and three OWF) near the inside of the dyke (Figure 1b, c). The average areas of the quadrats in artificial brackish marsh, paddy fields, and aquaculture ponds were 108.00 ± 5.58 ha, 32.53 ± 1.74 ha, and 8.50 ± 0.68 ha (mean ± standard error [*SE*]), respectively (Table S1).

Each field survey lasted for 3–4 days and was conducted after sunrise on sunny days. Two or three investigators conducted the field surveys during low tide and counted ducks using a spotting scope (20–60×) and binoculars (8×). To guarantee that all the ducks were counted, all quadrats were surveyed along the dykes, roads, or paddies. Each quadrat was scanned for at least 10 min, with no maximum time limit for completing a count; however, the counts were completed as rapidly as possible to avoid double counting birds (Xie, Zhang, Li, Ma, & Wang, 2019). All ducks were identified to species level, and all individuals in the survey area were counted. Ducks that only flew over the survey area were not included in the survey.

### 2.3 Satellite tracking

Ducks were captured using clap nets (22 × 5.4 m) in the capture pond at Chongming Dongtan in December 2018, as part of a banding program (Tang et al., 2019), with permission from the Agricultural Committee of Chongming District, Shanghai (Figure 1a). Twelve ducks (six Eastern spot‐billed ducks and six Mallards, the two most abundant duck species) were tagged from 16 to 19 December 2018 near the capture pond at Chongming Dongtan (Table S2). After routine biometric measurements, the ducks were weighed (to the nearest 0.1 g) using electronic scales, and heavier ducks (Eastern spot‐billed duck, male, 1,125.1–1125.4 g; female, 1,002.1–1,112.0 g; Mallard, male, 835.6–890.1 g; female, 835.1–1,020.1 g) were selected for the attachment of the GPS/GSM transmitters (HQBG 2715S, 17 g per tag, and HQBG 2512S, 14 g per tag; Hunan Global Messenger Technology Co. Ltd., China; Table S2). The transmitter was attached to the bird's back with a harness provided by HQXS and comprised <3% of the duck's body mass (Table S2; Hunan Global Messenger, 2019). Each bird remained caged for 30 min after attaching the transmitter to ensure that no abnormal behavior was observed, and the birds were then released near the capture pond (Kuang et al., 2019). The transmitters were set to record time, location, and instantaneous ground speed at 1‐ to 3‐hr intervals, depending on the battery conditions (solar insolation vs. built‐in battery). The behaviors and locations of the tagged ducks were initially monitored in the field for 2–3 days after release, to ensure that the recorded data were reasonable.

### 2.4 Landscape classification

The satellite image (path/row = 118/38) of our study area was downloaded from US Geological Survey (http://glovis.usgs.gov/) during the study period. We obtained high‐resolution (15 m) data using nearest neighbor diffusion to sharpen the multispectral and panchromatic data after clipping, radiometric calibration, geometric correction, and atmospheric correction (Kuang et al., 2019). We then used artificial visual interpretation and field surveys to classify land cover in ArcGIS 10.2 (ESRI) combined with Google Earth 17.3. The land cover in our study area (outer dotted line in Figure 1a, range, 468.65 km^2^) was classified into 11 categories related to duck habitat use based on field survey: mudflats, natural brackish marsh, artificial brackish marsh, paddy fields, upland fields, aquaculture ponds, roads, ditches, canals, woodland, and buildings.

### 2.5 Data analysis

#### 2.5.1 Duck abundance

Duck species and abundance recorded in the seven surveys were gathered for each habitat type (artificial brackish marsh, paddy fields, and aquaculture ponds) in the IWF and OWF sites. The average duck densities recorded over the seven surveys were compared between the IWF and OWF sites among the three habitats (Ma et al., 2007; Zou et al., 2016). The normality of duck abundance in each habitat and in relation to IWF/OWF was tested by the Shapiro–Wilk test. Differences in duck abundance among habitats and between IWF and OWF sites were analyzed by two‐way ANOVA. Duck densities in IWF and OWF sites in each habitat were compared using independent sample *t* tests. Tukey's honestly significant difference (HSD) test was used to conduct *post hoc* multiple comparisons if ANOVA indicated a significant difference in habitat type or in relation to wind farm presence.

#### 2.5.2 Duck distributions and overlap with wind turbines

We collected data from tagged ducks that stayed at Chongming Dongtan in the winter. Data were downloaded from the data service platform provided by the manufacturer (HQXS, 2019), and records with a positioning error <10 m were included in our study. We excluded data from the first 7 days after marking to avoid any effects of capture and tagging (Kuang et al., 2019), and excluded records from the last 7 days before the departure date of the spring migration because of potentially different habitat use and behaviors before migration (Papers et al., 2008). We defined migration as all locations >50 km away from the study area, and/or ducks moving in one direction at >20 km/hr (Chan et al., 2019). Movements with an instantaneous ground speed >5 km/hr were also discarded.

We divided the data into records obtained during the day and at night because of differences in distribution and habitat use by ducks during the day and at night (Krainyk et al., 2018; Parejo et al., 2019; Yetter et al., 2018). We defined day as the period between the beginning of civil dawn and the end of dusk at Chongming Dongtan, and night as the period from the end of civil dusk to the beginning of civil dawn on the next day. The times of civil dawn and dusk each day were derived using the package “maptools” (Bivand, 2019) in R 3.6.0 (R Core Team, 2019).

The habitat type of high‐resolution records was classified according to their land cover during the day and at night. The diversities of habitat types used by wintering ducks during the day and at night were also analyzed using the Shannon–Wiener index. Independent sample *t* tests were used to compare differences in habitats between day and night.

We estimated the overlaps between wintering ducks and wind turbines at Chongming Dongtan during the day and at night from December 2018 to March 2019 using the grid‐based method (Pearse, Brandt, & Krapu, 2016). We divided the study area into 500 × 500 m (0.25 km^2^) squares based on the distance between the turbines (average 500 m) at Chongming Dongtan. We then estimated the wintering duck locations (points) (composite and individual) in each 0.25‐km^2^ cell and determined the distribution of the wind turbines in the study area using the same grid method.

Overlap was determined as the percentage of cells with at least one duck location (true movement) and at least one wind turbine. However, very few ducks were located in wind turbine grid cells because the interval between records was at least 1 hr, potentially leading to an underestimation of the risk related to turbines. Based on tracking of individual movement (McDuie et al., 2019), we assumed that if two subsequent records were located on either side of a line of turbines, this indicated that the duck had crossed through the turbine area or flown around it. We therefore defined the expected overlap as the percentage of cells with at least one presumed duck crossing (potential movement) and the occurrence of at least one wind turbine. We defined overlap cells as cells with true (≥1 duck location) and potential (presumed duck crossings) duck movements and at least one wind turbine. We then assessed the observed and expected intensities of use (four levels: 1–5, 6–10, 10–20, and >20) in overlap cells by calculating the number of duck locations and presumed crossings, respectively.

We analyzed the differences between the percentages of observed and expected overlaps, and the average observed and expected intensity of use in overlap cells using the chi‐square tests.

#### 2.5.3 Duck habitat use based on satellite tracking

We assessed the effects of the wind farms on duck habitat use based on satellite tracking data, which could record duck movements during the day and at night at multiple scales (Zhang, Li, Yu, & Si, 2018). We did not use the field survey data to determine duck habitat use and selection because it was limited by survey site, habitat accessibility, and time (especially at night). In addition, the wintering home ranges of ducks were broad and covered complex landscapes.

We used composite 95% utilization distributions (available) and 50% core‐use area (used) to assess the habitat use versus habitat availability for wintering ducks at Chongming Dongtan (Meattey et al., 2019; Pearse et al., 2016). We reduced spatial autocorrelation of duck locations (points) by removing adjacent points within the study area using Spatial Analyst in ArcGIS 10.2 (ESRI), and retaining points (38.09% of total duck records) with a minimum separation distance between points of 100 m (Meattey et al., 2019). We did not compare Eastern spot‐billed ducks and Mallards because they have similar habitat use and selection in the wintering season (Behney, O’Shaughnessy, Eichholz, & Stafford, 2018). We established composite kernel‐based utilization distributions for the tagged ducks in the study area using a Gaussian kernel and least squares cross‐validation bandwidth estimator using Home Range Tools 2.0 in ArcGIS 10.2 to assess duck habitat use during the day and at night (Ma, Gan, Choi, & Li, 2014). Following Pearse et al. (2016), we investigated resource selection by quantifying and comparing habitat variables within the composite 95% utilization distributions (available) and 50% core‐use areas (used). We scored the points within 50% core‐use areas (used) as 1, and 95% utilization distributions (available) as 0 for creating models, and did not assess the overlap between used and available points.

Some studies have shown effects of wind turbines on ducks (Larsen & Guillemette, 2007; Loesch et al., 2013), and the distributions of wintering ducks may be influenced by distance to water, human activity, and habitat type (Loring et al., 2014; Meattey et al., 2019; Walker et al., 2013; Zhang et al., 2020; Zou et al., 2019). We selected four variables related to duck habitat use to model resource selection functions (RSFs; Meattey et al., 2019; Palumbo, Petrie, Schummer, Rubin, & Bonner, 2019; Pearse et al., 2016; Table S3).

The distance to the nearest wind turbine (km) was calculated as the distance from the duck point to the edge of the nearest wind turbine. Distance to water (m) was calculated based on visual interpretation as the distance from the duck point to the edge of a mudflat, natural brackish marsh, artificial brackish marsh, aquaculture pond, ditch, or canal that was covered with water. Motor vehicles passing through on the road and construction activity in the study area meant that human activity could be a major factor affecting duck habitat use at Chongming Dongtan during the day and at night. We therefore quantified the index of human activity in each 0.25‐km^2^ cell based on field surveys during the day and at night, separately, and duck locations (points) in one cell were defined by the same human disturbance. We scored the level of human activity as the summary of three metrics related to wintering duck activity (Table S4): maximum number of motorized vehicles passing through (0–3), distance to road (0–3), and various construction activities (0–3) (Pearse et al., 2016; Tripp, Lendemer, & McCain, 2019). We conducted four field surveys (once in the middle of each month) to record the number of motorized vehicles passing through each cell during the day and at night, separately, with each observation lasting for 10 min. We recorded the average number of visible motor vehicles and inquired about the times and duration of construction activities in each cell from 9:00–11:00 during the day and 21:00–23:00 at night from the construction workers. Distance to road was calculated based on the Euclidean distance between the cell and the edge of the road. Land cover at duck locations was defined as natural brackish marsh, artificial brackish marsh, paddy fields, and others (mudflat, upland field, ditch, canal) based on the overlapping between landscape classifications layer and each duck location (point). We assessed correlations between the variables using Pearson's product–moment correlations and checked for multicollinearity of variables using variance inflation factors (VIFs). Pairwise correlations among variables within samples throughout the study period did not exceed 0.7 and VIF values were ≤ 4. We therefore retained all the variables (Loring et al., 2014).

We estimated RSFs by mixed‐models logistic regression, with the time of records and bird ID as random effects, to avoid temporal autocorrelation and individual variability (Loring et al., 2014; Meattey et al., 2019; Pearse et al., 2016). A total of 32 models were created to describe the RSFs of wintering ducks at Chongming Dongtan during the day and at night. Candidate models were selected using Akaike's information criterion (AIC) and ranked using AIC_c_ differences (ΔAIC_c_) and AIC weights (*W_i_*) to estimate the relative likelihood of each candidate model (Burnham & Anderson, 2002). Competitive models were considered at ΔAIC_c_ ≤ 4.0 from the best‐performing model if they contained no uninformative parameters, and we selected the parameter coefficients from the most parsimonious model to calculate the RSFs. If the *W_i_* suggested no individual model was clearly the best (*W_i_* > 0.9), we used model averaging to provide model coefficients and variances (Anderson, Link, Johnson, & Burnham, 2001). Following Pearse et al. (2016), we employed the method of k‐fold cross‐validation to evaluate the model fit. Our data were divided into 10 k‐folds based on the Huberty (1994) rule of thumb, and we reported the average Spearman's correlation for the 10 iterations (Bélanger, Leblond, & Côté, 2019). All statistical analyses were conducted in R 3.6.0 (R Core Team, 2019), with the “glmulti” (Calcagno, 2019) and “MuMIn” (Bartoń, 2019) packages for model selection and averaging.

## 3 RESULTS

### 3.1 Duck abundance

We observed a total of 5,077 individuals from 12 species at the IWF site and 10,650 individuals from 15 species at the OWF site at Chongming Dongtan (Table 1). Duck density differed significantly among the habitat types (*p* < .01) and in relation to the wind farms (*p* < .001), according to two‐way ANOVA (Table 2).

**Table 1:** Duck records at sites inside (IWF) and outside wind farms (OWF) among the three habitats (ABM = artificial brackish marsh; PF = paddy fields; AP = aquaculture ponds) at Chongming Dongtan, China

Species	Scientific name	Duck ecological group	Habitat type			IWF	OWF	Total number of ducks	Relative abundance (%)
			ABM	PF	AP				
Eastern spot‐billed duck	*Anas zonorhyncha*	Dabbling duck	5,184	18	49	1,590	3,301	5,251	33.39
Mallard	*Anas platyrhynchos*	Dabbling duck	2,618		6	987	1637	2,624	16.68
Green‐winged teal	*Anas carolinensis*	Dabbling duck	638	365	151	345	909	1,254	7.97
Eurasian wigeon	*Mareca penelope*	Dabbling duck	439			8	431	439	2.79
Gadwall	*Mareca strepera*	Dabbling duck	614		14	56	572	628	3.99
Northern pintail	*Anas acuta*	Dabbling duck	649			66	583	649	4.13
Falcated duck	*Mareca falcata*	Dabbling duck	1,065		7	591	474	1,065	6.77
Northern shoveler	*Spatula clypeata*	Dabbling duck	1,009		6	280	736	1,016	6.46
Common pochard	*Aythya ferina*	Diving duck	2,574			727	1847	2,574	16.37
Baer's pochard	*Aythya baeri*	Diving duck	8		9		8	8	0.05
Greater scaup	*Aythya marila*	Diving duck	2				11	11	0.07
Tufted duck	*Aythya fuligula*	Diving duck	135			58	77	135	0.86
Smew	*Mergellus albellus*	Diving duck	66			6	60	66	0.42
Common merganser	*Mergus merganser*	Diving duck	5			3	2	5	0.03
Mandarin duck	*Aix galericulata*	Dabbling duck		2			2	2	0.01
Total duck species			14	3	7	12	15	15	–
Total duck individuals			15,006	485	236	5,077	10,650	15,727	1

Abbreviations: ABM, artificial brackish marsh, AP, aquaculture pond, IWF, inside wind farm sites, OWF, outside wind farm sites, PF, paddy field.

**Table 2:** Results of two‐way ANOVA of habitat type and wind farm presence on duck abundance

Factor	*df*	F	*P*
Abundance			
Habitat type	2	11.367	**0.001**
Wind farm	1	21.717	**<0.001**
Habitat type × wind farm	2	2.662	0.097

**Figure 2:**
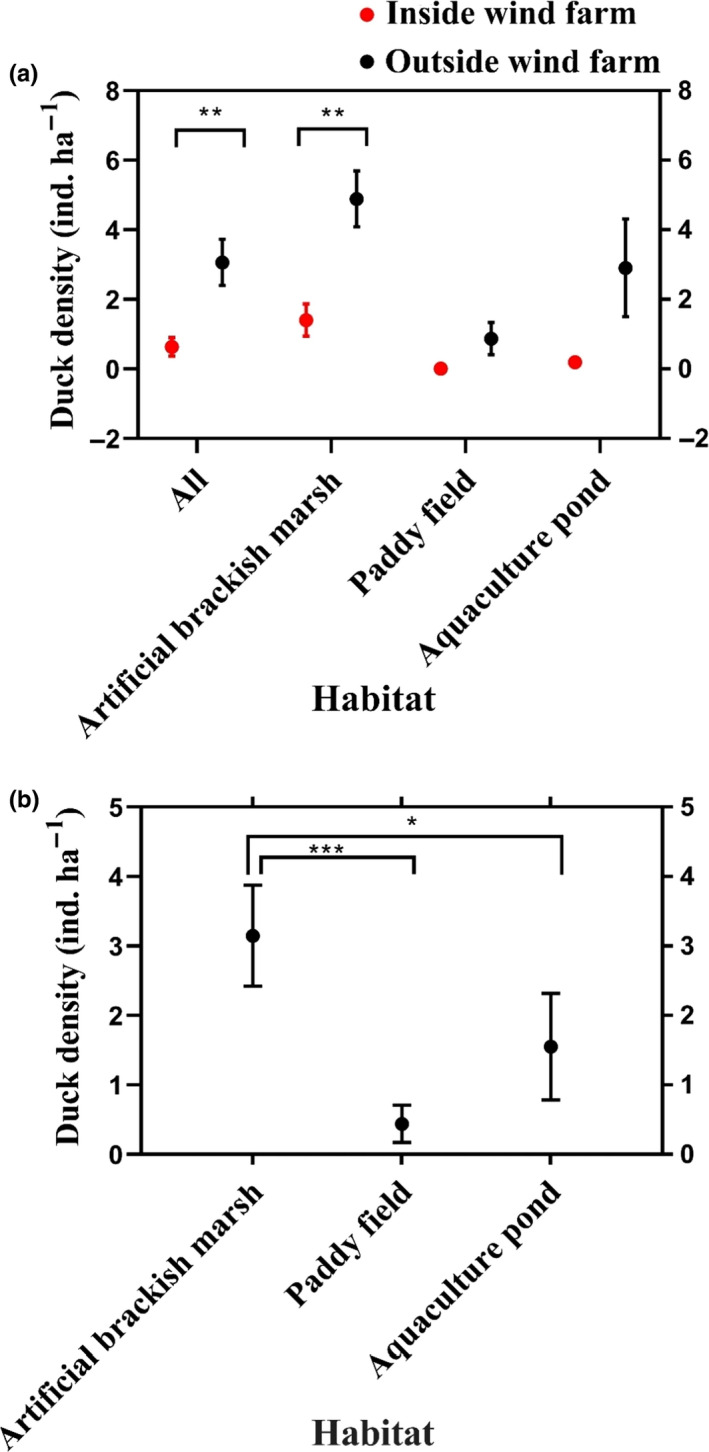
Duck densities at sites inside wind farms (IWF) and outside wind farms (OWF) overall and in each habitat (a) and among three habitats (b) at Chongming Dongtan in the Yangtze River mouth, China. Results shown as mean ± standard error of duck density (ind/ha). Horizontal lines indicate significant differences in duck density between artificial brackish marsh and paddy fields. ***p < .001; **p < .01; *p < .05

Mean duck density (±*SE*) at the IWF sites (0.64 ± 0.27 ind/ha) was significantly lower than at the OWF sites (3.06 ± 0.66 ind/ha) (*p < *.05) (Figure 2a). Duck density in artificial brackish marsh was also significantly higher at the OWF (4.89 ± 0.80 ind/ha) compared with the IWF site (1.41 ± 0.46 ind/ha) (*p < *.05) (Figure 2a). According to Tukey's HSD test, duck density was significantly higher in artificial brackish marsh (3.15 ± 0.73 ind/ha) compared with paddy fields (0.44 ± 0.27 ind/ha) (*p < *.001) and aquaculture ponds (1.55 ± 0.77 ind/ha) (*p < *.05) (Figure 2b).

### 3.2 Duck distributions and overlap with wind farm

Three tagged Mallards lost their signals on the fourth day of tracking, and data for a total of nine ducks (six Eastern spot‐billed ducks and three Mallards) were therefore analyzed in this study (Table S2). These birds provided 1,918 high‐resolution records at Chongming Dongtan, including 1,998 at night and 899 during the day. The mean number of locations was 240 ± 40 per duck (range: 159–288).

The distributions of wintering ducks were relatively concentrated during the day compared with at night. The main habitat types used by the tagged ducks during the day were artificial brackish marsh (70.98%), natural brackish marsh (27.48%), and paddy fields (1.53%), and the main habitat types at night were paddy fields (50.00%), natural brackish marsh (27.93%), and artificial brackish marsh (8.78%). The diversity of habitats used was significantly higher at night (1.09 ± 0.35) than during the day (0.38 ± 0.31; *p* < .001).

**Figure 3:**
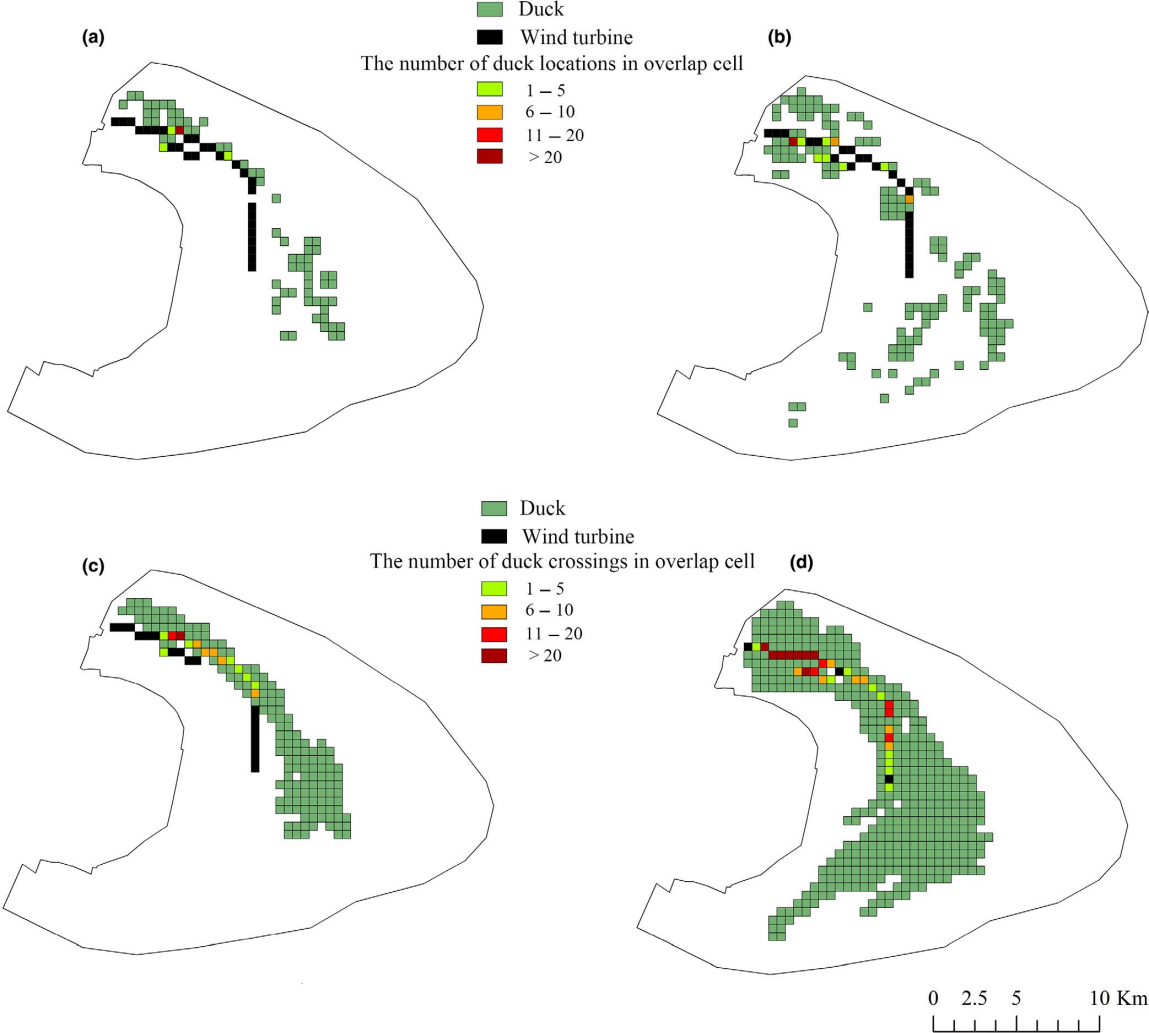
Observed overlaps between duck locations and wind turbines during the day (a) and at night (b), and expected overlaps between duck crossings and wind turbines during the day (c) and at nigh*t (d) Chongming Dongtan, China, from December 2018 to March 2019. Unit of analysis was 0.25 km^2^ (0.5 × 0.5 km) grid cells*

The overlap rate during the day (Figure 3a, c) was lower than at night (Figure 3b, d). Ducks locations appeared in 10.78% of grid cells within the study area at night (Figure 3b) and only 5.21% of grid cells during the day (Figure 3a), while presumed duck crossings appeared in 32.18% of grid cells within the study area at night (Figure 3d), and 13.12% of grid cells during the day (Figure 3c). Turbines occurred in 2.35% of grid cells within study area, and 28.13% of turbine cells overlapped with cells appeared by duck locations at night (Figure 3b), compared with only 12.50% of overlap during the day (Figure 3a). However, based on the presumed duck crossings, 90.63% of turbine cells overlapped with cells appeared by duck crossings at night (Figure 3d), compared with 43.75% of overlap during the day (Figure 3c). There was a significant difference between the percentages of observed and expected overlaps based on duck locations during the day (χ^2^_day_ = 62.38, *p* < .01) and at night (χ^2^_night_ = 167.29, *p* < .01), and a significant difference in average observed and expected intensities of use based on duck crossings in overlap cells during the day (χ^2^_day_ = 19.88, *p* < .01) and at night (χ^2^_night_ = 17.27, *p* < .01).

### 3.3 Effect of wind farms on duck habitat use based on satellite tracking

**Figure 4:**
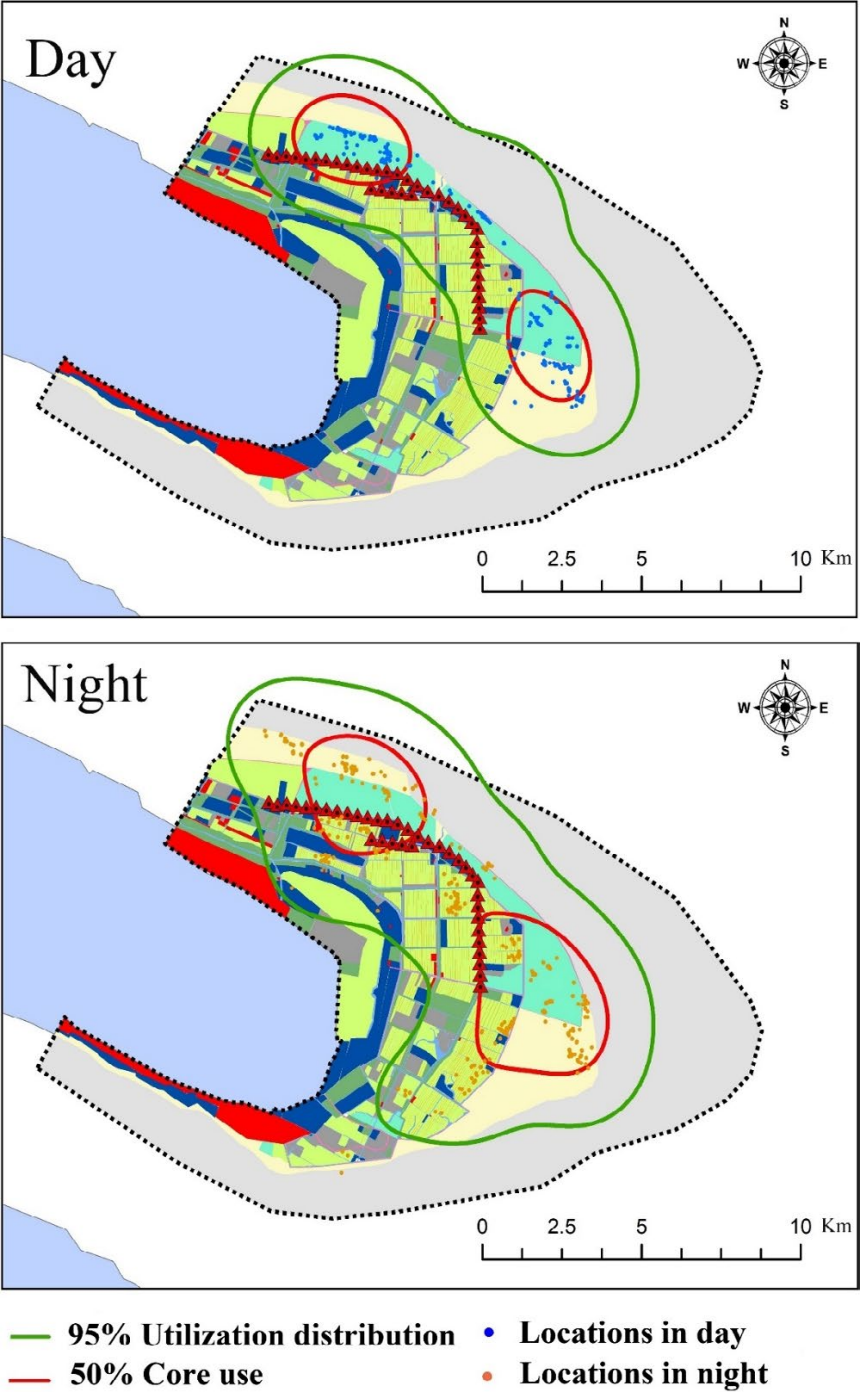
Wintering duck composite kernel‐based 50% core‐use (brown) areas and 95% utilization distributions (green) during the day (top) and at night (bottom) at Chongming Dongtan, China, from December 2018 to March 2019

The area of kernel‐based utilization distribution showed that the composite 50% core‐use areas and 95% utilization distributions during the day were 35.09 km^2^ and 153.99 km^2^, and the equivalent areas at night were 55.46 km^2^ and 233.00 km^2^, respectively (Figure 4).

Based on the average of the top two models during the day and at night, duck core‐use areas were positively associated with distance to wind turbines, relative to utilization distributions. The top two models during the day were selected based on AIC_C_ (△AIC_c_ < 4) (Table S5), both including distance to wind turbine, distance to water, and index of human activity. Spearman's correlation coefficients based on cross‐validation were robust (*r^‐^_s_* = 0.72). An average of the top two models showed that ducks at Chongming Dongtan selected areas with less human activity (estimate = −3.51, 95% confidence interval (CI) [−4.52, −2.49]) and far from wind turbines (estimate = 1.94, 95% CI [0.98, 2.90]) (Table 3). Compared with natural brackish marsh, ducks showed strong avoidance of paddy fields (estimate = −3.52, 95% CI [−6.01, −0.29]) and slight avoidance of artificial brackish marsh (Table 3).

**Table 3:** Average of top two models during the day and at night for resource selection for wintering ducks at Chongming Dongtan, China, from December 2018 to March 2019

Period	Variable	Estimate	Lower 95% CI	Upper 95% CI
Day	Intercept	22.10	14.04	30.17
	Distance to wind turbine	1.94	0.98	2.90
	Distance to water	−0.01	−0.01	−0.01
	Index of human activity	−3.51	−4.52	−2.49
	Land cover			
	Artificial brackish marsh	−1.35	−4.47	1.77
	Paddy fields	−3.52	−6.01	−0.29
	Other	−31.90	−5,863,816.00	5,863,752.00
Night	Intercept	−4.86	−6.58	−3.13
	Distance to wind turbine	0.58	0.32	0.84
	Distance to water	0.01	0.00	0.01
	Index of human activity	0.06	−0.19	0.55
	Land cover			
	Artificial brackish marsh	1.17	−0.13	2.47
	Paddy fields	2.79	1.50	4.09
	Other	16.82	−0.09	920.84

The top two models at night were selected using △AIC_C_ < 4 (Table S5). Both included distance to wind turbine, index of human activity, and land cover as variables (Table 3). Spearman's correlation coefficients based on cross‐validation were robust (*r^‐^_s_* = 0.72). An average of the top two models showed that ducks selected areas far from wind turbines (estimate = 0.58, 95% CI [0.32, 0.84]), with strong selection of paddy fields (estimate = 2.79, 95% CI [1.50, 4.09]) compared with natural brackish marsh (Table 3). Coefficients for distance to water and index of human activity showed weak selection of areas away from water and with less human activity.

## 4 DISCUSSION

In the context of the rapid development of onshore wind farms, there is an urgent need globally to evaluate the impact of these wind farms on waterbirds in coastal wetlands, with the aim of mitigating their indirect effects. In this study, we investigated the impacts of wind farms on duck abundance, distribution, and habitat use at Chongming Dongtan using field surveys and satellite tracking. The results revealed that wintering ducks tended to avoid wind turbines areas at Chongming Dongtan, and highlighted the importance of assessing the landscape characteristics of proposed construction sites for onshore wind farms in coastal wetlands along the East Asian–Australasian Flyway.

### 4.1 Effects of wind farms on duck abundance

Many studies have shown that habitat conditions and anthropogenic impacts can influence the abundance of waterbirds (Shaffer & Buhl, 2016; Zou et al., 2019), and ducks, as the dominant ecological taxon at Chongming Dongtan in winter (Zou et al., 2016), could respond to wind farm flexible in population or communication level. Wind farms, as anthropogenic facilities, may affect duck density as a result of habitat displacement due to behavioral changes caused by wind turbine vibration or habitat occupancy, as reported in the Prairie Pothole Region (Loesch et al., 2013). The wind turbines at Chongming Dongtan were located at the edges of paddy fields and artificial brackish marsh, which may cause the ducks to change their behavior and redistribute to the OWF site. Paddy fields and artificial brackish marsh were the main habitat types for ducks, and the ducks need to move daily between paddy fields and artificial brackish marsh for feeding and resting (Parejo et al., 2019). Several previous studies revealed that avoidance of land‐based wind energy developments by ducks did not imply complete abandonment of an area, but rather reduced use of a site with the continued presence of ducks at wind farm sites, but at reduced densities (Loesch et al., 2013). This might help explain the lower density of ducks at IWF sites to some extent. Although bird abundance might be influenced by edge effects (Khamcha et al., 2018) and human disturbance (Zou et al., 2019), we believe that the edge effect would not have a significant impact on duck abundance, and there would have been no significant difference in human disturbance (mainly human activity) among the quadrats in the current study. The selected quadrats in each habitat in IWF and OWF sites were relatively uniformly sized, and ducks usually inhabited the center of artificial brackish marsh areas and aquaculture ponds, which would be less prone to edge effects. The quadrats in each habitat were also located near to or along the dyke, and given that the road was constructed on the dyke, this would lead to similar levels of disturbance among the quadrats in each habitat during the day. However, we recommend that further long‐term, large‐scale waterfowl studies should be conducted along the East Asian–Australasian Flyway to inform wind energy development projects and to reduce their impacts on duck abundance.

Duck densities in artificial brackish marsh was significantly higher than in compared with aquaculture ponds and paddy fields in our study, possibly due to different levels of human activity, water levels, and food resources among the three habitats. These results were in accord with the satellite tracking outcomes during the day. There was frequent human activity in the aquaculture ponds and paddy fields at Chongming Dongtan during the day, including agricultural activities (Xie et al., 2019) and motorized vehicles, which could have a negative effect on duck abundance. In contrast, artificial brackish marsh, as a restoring wetland, offered stable water levels and low human activity (Kuang et al., 2019), potentially making it a better wintering habitat for ducks.

### 4.2 Duck distributions and overlap with wind farms

The distribution of ducks at Chongming Dongtan differed significantly between day and night from December 2018 to March 2019, which were largely in line with the reported habitat use by Mallards at an autumn stopover on the Northwest European Flyway (Bengtsson et al., 2014), and by Australian wood ducks (*Chenonetta jubata*) in an agricultural landscape (Fernández‐Bellon et al., 2019), and consistent with duck distribution patterns (Eastern spot‐billed ducks and Mallards) at Chongming Dongtan (Jiang et al., 2007). In addition, Ma et al. (2004) found similar results in that inland aquaculture ponds provided more suitable habitats for ducks compared with brackish marsh at Chongming Island in winter, with local movement between them. We conjectured that the tidal pattern and duck activity rhythm might be factors driving the different distributions between day and night.

Although the current study only had small sample sizes and only included two duck species, Eastern spot‐billed ducks and Mallards are the dominant duck species at Chongming Dongtan, accounting for 50.07% of the total duck population according to our field surveys (Table 1; Eastern spot‐billed duck, 33.39% of total duck population; Mallard, 16.68% of total duck population), and also representing common duck species along the East China Coast and East Asian–Australasian Flyway (Bai et al., 2015; Cao et al., 2008). Our results based on tagging these two typical species along the East China Coast emphasized the different distributions of ducks in coastal wetlands during the day and at night, with possible implications for distribution changes or local movements between these periods. Ducks fitted with tracking equipment may be adversely affected, depending on their individual body mass and impacts on the hunting risk (Barron, Brawn, & Weatherhead, 2010; Ma, 2013); however, we did not consider these variables to be important in the current study because all the equipped birds met the body mass threshold for tracking equipment (<3%), and because ducks at Chongming Dongtan are at low risk of being hunted because of many protected areas in this region (Choi et al., 2019).

In our study, overlap rate between ducks and wind turbines during the day was lower than at night at Chongming Dongtan. The habitat type around wind turbines might help to explain this difference in overlap rates. Most wind turbines in Chongming Dongtan (~62.50% of wind turbines adjacent to paddy fields) were located in grid cells containing paddy fields, which were preferred by ducks at night, highlighting the importance of paddy fields for ducks in coastal wetlands in the winter.

The overlap rates between wind farms and ducks might have been underestimated, given that wind turbines occurred in very few grid cells and the number of tagged individuals was limited. However, chi‐square tests indicated significant differences between percent of the observed and expected overlaps and observed and expected intensities of use in overlap cells during the day and at night, suggesting that the overlaps between wind turbines and ducks were actually higher. This can be explained by the recording interval of the tracking equipment and duck movement ability. We set the recording time to 1 hr based on the battery conditions (solar insolation vs. built‐in battery), which meant that we missed some movement data, while the strong movement ability of ducks would allow them to cross each cell easily (500 × 500 m). We therefore found a lower overlap rate and intensity of use based on duck locations (observed) compared with that based on crossings (expected).

The result of duck abundance field surveys showed that 5,077 of duck individuals (approximately 32.28%) at site indise wind farms (Table 1), indicating that the habitats around wind farms were of great interest to wintering ducks at Chongming Dongtan. We therefore suggest that habitats including artificial wetlands (aquaculture ponds) and paddy fields adjoining natural wetlands should be avoided when planning future wind energy developments in coastal wetlands.

### 4.3 Effects of wind farms on duck habitat use based on satellite tracking

Our study showed that ducks tended to select core areas with low human activity during the day and far from wind turbines both during the day and at night. Numerous previous studies have identified human activity as an important environmental factor affecting waterbird distributions (Cornelius, Navarrete, & Marquet, 2001; Peters & Otis, 2007; Zou et al., 2019), because waterbirds tend to select sites away from human settlements (especially roads and buildings). During the day, the index of human activity was a crucial negative‐impact factor influencing habitat use of duck in this study, suggesting frequent and intensive human activities at Chongming Dongtan during the day. To the best of our knowledge, ducks are alert waterbirds in their natural environment and would thus be sensitive to human activity (construction activities and vehicles along the roads; Mo et al., 2017; Yang et al., 2013). Ducks tended to inhabit areas far from wind turbines at Chongming Dongtan, both during the day and at night, in accord with wintering common eiders (*Somateria mollissima*) in offshore areas (Desholm & Kahlert, 2005; Larsen & Guillemette, 2007). This habitat use in response to wind farms indicated that ducks would avoid wind turbines. Although we did not carry out a detailed exploration of the mechanisms by which wind turbines affected ducks in this study, the long‐term effects of wind farms on duck habitat use could lead to functional habitat loss.

Onshore wind farms have been constructed or proposed at many sites along the East China Coast (e.g., Yancheng, Rudong, Dongtai, and Qidong), especially in artificial wetlands (aquaculture ponds) and paddy fields adjoining natural wetlands and/or wetland reserves, to satisfy energy demands (Global Wind Energy Council, 2019). However, large‐scale surveys identified the East China Coast as a wintering ground for ducks from northern China, Mongolia, and central and eastern Siberia, and as a key component of the East Asian–Australasian Flyway (Cao et al., 2008; Jackson et al., 2019). Although the National Forestry and Grassland Administration of China has issued guidelines about the use of forested land for wind farm projects (State Forestry & Grassland Administration of China, 2019), further research is needed regarding the planning and management of onshore wind farms in relation to waterbirds in coastal wetlands. Although direct collision risk is of minimal concern for ducks (Meattey et al., 2019), the effects of displacement and obstruction could have multiple effects on the birds’ ability to respond to seasonally dynamic habitat quality and on the utilization efficiency of the entire wintering area. The results in terms of the abundance, distributions, and habitat use of dominant duck species around wind farms in our study suggested that ducks would avoid onshore wind farm areas in coastal wetlands, and the landscape of paddy fields and artificial wetlands adjoining natural wetlands along the coast was crucial to ducks. There are currently large area and many key sites (e.g., Guanyu Coast, Linhong Estuary, Xiuzhenhe, Liezikou, Tiaozini, Rudong Coast, Dongling Coast) similar to Chongming Dongtan (Duan et al., 2020), consisting of long stretches of continuous mudflat, and subcoastal wetlands mainly comprising aquaculture ponds, salt pans, and paddy fields along the East China Coast including the East Asian–Australasian Flyway (Cao et al., 2008). We therefore recommend that the potential effects of different land cover on waterbirds should be considered in relation to future onshore wind farm developments in coastal wetlands.

## CONFLICT OF INTEREST

None declared.

## AUTHOR CONTRIBUTION

**Shanshan Zhao:** Formal analysis (lead); Investigation (lead); Software (lead); Writing‐original draft (lead). **Huan Xu:** Investigation (supporting). **Ningning Song:** Investigation (supporting). **Zhenghuan Wang:** Data curation (supporting); Resources (supporting). **Ben Li:** Conceptualization (equal); Data curation (equal); Formal analysis (equal); Funding acquisition (supporting). **Tianhou Wang:** Conceptualization (lead); Funding acquisition (lead); Methodology (lead); Project administration (lead).

## Supporting information

Table S1‐S5

## Data Availability

Relevant data will be available via Dryad: https://doi.org/10.5061/dryad.95x69p8hd.

## References

[ece36701-bib-0001] Anderson, D. R. , Link, W. A. , Johnson, D. H. , & Burnham, K. P. (2001). Suggestions for presenting the results of data analyses. Journal of Wildlife Management, 65(3), 373 10.2307/3803088

[ece36701-bib-0002] Aschwanden, J. , Stark, H. , Peter, D. , Steuri, T. , Schmid, B. , & Liechti, F. (2018). Bird collisions at wind turbines in a mountainous area related to bird movement intensities measured by radar. Biological Conservation, 220, 228–236. 10.1016/j.biocon.2018.01.005

[ece36701-bib-0003] Bai, Q. , Chen, J. , Chen, Z. , Dong, G. , Dong, J. , Dong, W. , … Zeng, X. (2015). Identification of coastal wetlands of international importance for waterbirds: A review of China Coastal Waterbird Surveys 2005–2013. Avian Research, 6(1), 1–16. 10.1186/s40657-015-0021-2

[ece36701-bib-0004] Bamford, M. , Watkins, D. , Bancroft, W. , Tischler, G. , & Wahl, J. (2008). Migratory shorebirds of the East Asian‐Australasian Flyway: Population estimates and internationally important sites. Canberra, ACT: Wetlands International ‐ Oceania.

[ece36701-bib-0005] Barron, D. G. , Brawn, J. D. , & Weatherhead, P. J. (2010). Meta‐analysis of transmitter effects on avian behaviour and ecology. Methods in Ecology and Evolution, 1(2), 180–187. 10.1111/j.2041-210x.2010.00013.x

[ece36701-bib-0006] Barter, M. , & Wang, T. (1990). Can waders fly non‐stop from Australia to China? Stilt, 17, 36–39.

[ece36701-bib-0007] Bartoń, K. (2019). MuMIn: Multi‐Model Inference. Retrieved from https://cran.r‐project.org/web/packages/MuMIn/index.html

[ece36701-bib-0008] Behney, A. C. , O’Shaughnessy, R. , Eichholz, M. W. , & Stafford, J. D. (2018). Indirect risk effects reduce feeding efficiency of ducks during spring. Ecology and Evolution, 8(2), 961–972. 10.1002/ece3.3714 29375770 PMC5773304

[ece36701-bib-0009] Bélanger, É. , Leblond, M. , & Côté, S. D. (2019). Habitat selection and population trends of the Torngat Mountains caribou herd. Journal of Wildlife Management, 83(2), 379–392. 10.1002/jwmg.21583

[ece36701-bib-0010] Bengtsson, D. , Avril, A. , Gunnarsson, G. , Elmberg, J. , Söderquist, P. , Norevik, G. , … Waldenström, J. (2014). Movements, home‐range size and habitat selection of Mallards during autumn migration. PLoS One, 9(6), e100764 10.1371/journal.pone.0100764 24971887 PMC4074106

[ece36701-bib-0011] Bivand, R. (2019). maptools: Tools for Handling Spatial Objects. Retrieved from http://maptools.r‐forge.r‐project.org/

[ece36701-bib-0012] Bradbury, G. , Trinder, M. , Furness, B. , Banks, A. N. , Caldow, R. W. G. , & Hume, D. (2014). Mapping Seabird Sensitivity to offshore wind farms. PLoS One, 9(9), e106366 10.1371/journal.pone.0106366 25210739 PMC4161323

[ece36701-bib-0013] Burnham, K. , & Anderson, D. (2002). Model selection and multimodel inference, a practical information‐theoretic approach. New York, NY: Springer.

[ece36701-bib-0014] Calcagno, V. (2019). glmulti: Model Selection and Multimodel Inference Made Easy. Retrieved from https://cran.r‐project.org/web/packages/glmulti/index.html

[ece36701-bib-0015] Cao, L. , Barter, M. , & Lei, G. (2008). New Anatidae population estimates for eastern China: Implications for current flyway estimates. Biological Conservation, 141(9), 2301–2309. 10.1016/j.biocon.2008.06.022

[ece36701-bib-0016] Cao, L. , Zhang, Y. , Barter, M. , & Lei, G. (2010). Anatidae in eastern China during the non‐breeding season: Geographical distributions and protection status. Biological Conservation, 143(3), 650–659. 10.1016/j.biocon.2009.12.001

[ece36701-bib-0017] Chan, Y.‐C. , Tibbitts, T. L. , Lok, T. , Hassell, C. J. , Peng, H.‐B. , Ma, Z. , … Piersma, T. (2019). Filling knowledge gaps in a threatened shorebird flyway through satellite tracking. Journal of Applied Ecology, 56(10), 2305–2315. 10.1111/1365-2664.13474

[ece36701-bib-0018] Choi, C. Y. , Battley, P. F. , Potter, M. A. , Rogers, K. G. , & Ma, Z. (2015). The importance of Yalu Jiang coastal wetland in the north Yellow Sea to Bar‐tailed Godwits Limosa lapponica and Great Knots Calidris tenuirostris during northward migration. Bird Conservation International, 25(1), 53–70. 10.1017/S0959270914000124

[ece36701-bib-0019] Choi, C.‐Y. , Peng, H.‐B. , He, P. , Ren, X.‐T. , Zhang, S. , Jackson, M. V. , … Ma, Z. (2019). Where to draw the line? Using movement data to inform protected area design and conserve mobile species. Biological Conservation, 234, 64–71. 10.1016/j.biocon.2019.03.025

[ece36701-bib-0020] Cornelius, C. , Navarrete, S. A. , & Marquet, P. A. (2001). Effects of human activity on the structure of coastal marine bird assemblages in Central Chile. Conservation Biology, 15(5), 1396–1404. 10.1046/j.1523-1739.2001.00163.x

[ece36701-bib-0021] Dahl, E. L. , Bevanger, K. , Nygård, T. , Røskaft, E. , & Stokke, B. G. (2012). Reduced breeding success in White‐tailed Eagles at Smøla windfarm, western Norway, is caused by mortality and displacement. Biological Conservation, 145(1), 79–85. 10.1016/j.biocon.2011.10.012

[ece36701-bib-0022] Desholm, M. , & Kahlert, J. (2005). Avian collision risk at an offshore wind farm. Biology Letters, 1(3), 296–298. 10.1098/rsbl.2005.0336 17148191 PMC1617151

[ece36701-bib-0023] Dhunny, A. Z. , Allam, Z. , Lobine, D. , & Lollchund, M. R. (2019). Sustainable renewable energy planning and wind farming optimization from a biodiversity perspective. Energy, 185, 1282–1297. 10.1016/j.energy.2019.07.147

[ece36701-bib-0024] Dierschke, V. , Furness, R. W. , & Garthe, S. (2016). Seabirds and offshore wind farms in European waters: Avoidance and attraction. Biological Conservation, 202, 59–68. 10.1016/j.biocon.2016.08.016

[ece36701-bib-0025] Dohm, R. , Jennelle, C. S. , Garvin, J. C. , & Drake, D. (2019). A long‐term assessment of raptor displacement at a wind farm. Frontiers in Ecology and the Environment, 17(8), 1–6. 10.1002/fee.2089

[ece36701-bib-0026] Duan, H. , Xia, S. , Jackson, M. V. , Zhao, N. , Liu, Y. U. , Teng, J. , … Shi, J. (2020). Identifying new sites of significance to waterbirds conservation and their habitat modification in the Yellow and Bohai Seas in China: Waterbird conservation planning in China. Global Ecology and Conservation, 22, e01031 10.1016/j.gecco.2020.e01031

[ece36701-bib-0027] Fernández‐Bellon, D. , Wilson, M. W. , Irwin, S. , & O’Halloran, J. (2019). Effects of development of wind energy and associated changes in land use on bird densities in upland areas. Conservation Biology, 33(2), 413–422. 10.1111/cobi.13239 30346052

[ece36701-bib-0028] Global Wind Energy Council (2019). Global wind report. Retrieved from https://gwec.net/

[ece36701-bib-0029] Gómez‐Catasús, J. , Garza, V. , & Traba, J. (2018). Wind farms affect the occurrence, abundance and population trends of small passerine birds: The case of the Dupont’s lark. Journal of Applied Ecology, 55(4), 2033–2042. 10.1111/1365-2664.13107

[ece36701-bib-0030] Graff, B. J. , Jenks, J. A. , Stafford, J. D. , Jensen, K. C. , & Grovenburg, T. W. (2016). Assessing spring direct mortality to avifauna from wind energy facilities in the Dakotas. Journal of Wildlife Management, 80(4), 736–745. 10.1002/jwmg.1051

[ece36701-bib-0031] Guillemette, M. , & Larsen, J. K. (2002). Postdevelopment experiments to detect anthropogenic disturbances: The case of sea ducks and wind parks. Ecological Applications, 12(3), 868–877. 10.2307/3060995

[ece36701-bib-0032] He, Z. , Xu, S. , Shen, W. , Long, R. , & Yang, H. (2016). Overview of the development of the Chinese Jiangsu coastal wind‐power industry cluster. Renewable and Sustainable Energy Reviews, 57, 59–71. 10.1016/j.rser.2015.12.187

[ece36701-bib-0033] Hötker, H. (2017). Birds: Displacement In Perrow M. R. (Ed.), Wildlife and wind farms, conflicts and solutions. Onshore: Potential Effects, Vol. 1 (pp. 157–190). Exeter, UK: Pelagic Publishing.

[ece36701-bib-0034] HQXS (2019). Wild Animal Micro Tracker operation instruction. Version 3.02. Retrieved from http://www.hqxs.net

[ece36701-bib-0035] Huberty, C. J. (1994). Applied discriminant analysis. New York, NY: Wiley Interscience.

[ece36701-bib-0036] Hunan Global Messenger Technology Co., Ltd Satellite Tracker Positional Accuracy Specification. Retrieved from http://www.hqxs.net/UploadImg/ServerFile/Server_20180927173636.pdf

[ece36701-bib-0037] Iwamura, T. , Possingham, H. P. , Chadès, I. , Minton, C. , Murray, N. J. , Rogers, D. I. , … Fuller, R. A. (2013). Migratory connectivity magnifies the consequences of habitat loss from sea‐level rise for shorebird populations. Proceedings of the Royal Society B: Biological Sciences, 280(1761), 20130325 10.1098/rspb.2013.0325 PMC3652437 23760637

[ece36701-bib-0038] Jackson, M. V. , Carrasco, L. R. , Choi, C.‐Y. , Li, J. , Ma, Z. , Melville, D. S. , … Fuller, R. A. (2019). Multiple habitat use by declining migratory birds necessitates joined‐up conservation. Ecology and Evolution, 9(5), 2505–2515. 10.1002/ece3.4895 30891196 PMC6405493

[ece36701-bib-0039] Jiang, S. , Ge, Z. , Pei, E. , Xu, X. , Sang, L. , & Wang, T. (2007). Waterfowl Nocturnal Behavior at the Artificial Wetlands behind the Chongming Dongtan Seawall in Winter. Chinese Journal of Zoology, 42(6), 21–27.

[ece36701-bib-0040] Kelsey, E. C. , Felis, J. J. , Czapanskiy, M. , Pereksta, D. M. , & Adams, J. (2018). Collision and displacement vulnerability to offshore wind energy infrastructure among marine birds of the Pacific Outer Continental Shelf. Journal of Environmental Management, 227, 229–247. 10.1016/j.jenvman.2018.08.051 30195148

[ece36701-bib-0041] Khamcha, D. , Corlett, R. T. , Powell, L. A. , Savini, T. , Lynam, A. J. , & Gale, G. A. (2018). Road induced edge effects on a forest bird community in tropical Asia. Avian Research, 9(1), 1–13. 10.1186/s40657-018-0112-y

[ece36701-bib-0042] Krainyk, A. , Finger, R. S. , Ballard, B. M. , Merendino, M. T. , Wester, D. B. , & Terry, R. H. (2018). Habitat selection by Mottled Duck broods. Wildfowl, 68, 104–122.

[ece36701-bib-0043] Kuang, F. , Wu, W. , Ke, W. , Ma, Q. , Chen, W. , Feng, X. , … Ma, Z. (2019). Habitat use by migrating Whimbrels (*Numenius phaeopus*) as determined by bio‐tracking at a stopover site in the Yellow Sea. Journal of Ornithology, 160(4), 1109–1119. 10.1007/s10336-019-01683-6

[ece36701-bib-0044] Kunz, T. H. , Arnett, E. B. , Cooper, B. M. , Erickson, W. P. , Larkin, R. P. , Mabee, T. , … Szewczak, J. M. (2007). Assessing impacts of wind‐energy development on nocturnally active birds and bats: A guidance document. Journal of Wildlife Management, 71(8), 2449–2486. 10.2193/2007-270

[ece36701-bib-0045] Kuvlesky, W. P. , Brennan, L. A. , Morrison, M. L. , Boydston, K. K. , Ballard, B. M. , & Bryant, F. C. (2007). Wind energy development and wildlife conservation: Challenges and Opportunities. Journal of Wildlife Management, 71(8), 2487–2498. 10.2193/2007-248

[ece36701-bib-0047] Larsen, J. K. , & Guillemette, M. (2007). Effects of wind turbines on flight behaviour of wintering Common Eiders: Implications for habitat use and collision risk. Journal of Applied Ecology, 44, 516–522. 10.1111/j.1365-2664.2007.1303.x

[ece36701-bib-0048] LeBeau, C. W. , Johnson, G. D. , Holloran, M. J. , Beck, J. L. , Nielson, R. M. , Kauffman, M. E. , … McDonald, T. L. (2017). Greater sage‐grouse habitat selection, survival, and wind energy infrastructure. Journal of Wildlife Management, 81(4), 690–711. 10.1002/jwmg.21231

[ece36701-bib-0049] Li, B. , Yuan, X. , Chen, M. , Bo, S. , Xia, L. , Guo, Y. U. , … Wang, T. (2020). How to strive for balance of coastal wind energy development with waterbird conservation in the important coastal wetlands, a case study in the Chongming Islands of East China. Journal of Cleaner Production, 263, 121547 10.1016/j.jclepro.2020.121547

[ece36701-bib-0050] Loesch, C. R. , Walker, J. A. , Reynolds, R. E. , Gleason, J. S. , Niemuth, N. D. , Stephens, S. E. , & Erickson, M. A. (2013). Effect of wind energy development on breeding duck densities in the Prairie Pothole Region. Journal of Wildlife Management, 77(3), 587–598. 10.1002/jwmg.481

[ece36701-bib-0051] Loring, P. H. , Paton, P. W. C. , Osenkowski, J. E. , Gilliland, S. G. , Savard, J. P. L. , & McWilliams, S. R. (2014). Habitat use and selection of Black Scoters in southern New England and siting of offshore wind energy facilities. Journal of Wildlife Management, 78(4), 645–656. 10.1002/jwmg.696

[ece36701-bib-0052] Ma, Z. (2013). Tracking the migration of birds. Science (KEXUE), 65(1), 19–22.

[ece36701-bib-0053] Ma, Z. , Gan, X. , Choi, C. , Jing, K. , Tang, S. , Li, B. , & Chen, J. (2007). Wintering bird communities in newly‐formed wetland in the Yangtze River estuary. Ecological Research, 22(1), 115–124. 10.1007/s11284-006-0193-7

[ece36701-bib-0054] Ma, Z. , Gan, X. , Choi, C. Y. , & Li, B. (2014). Effects of invasive cordgrass on presence of marsh grassbird in an area where it is not native. Conservation Biology, 28(1), 150–158. 10.1111/cobi.12172 24405105

[ece36701-bib-0055] Ma, Z. , Li, B. , Zhao, B. , Jing, K. , Tang, S. , & Chen, J. (2004). Are artificial wetlands good alternatives to natural wetlands for waterbirds? ‐ A case study on Chongming Island. China. Biodiversity and Conservation, 13(2), 333–350. 10.1023/B:BIOC.0000006502.96131.59

[ece36701-bib-0056] Ma, Z. , Wang, Y. , Gan, X. , Li, B. , Cai, Y. , & Chen, J. (2009). Waterbird population changes in the wetlands at Chongming dongtan in the Yangtze river estuary. China. Environmental Management, 43(6), 1187–1200. 10.1007/s00267-008-9247-7 19139954

[ece36701-bib-0057] Marques, A. T. , Santos, C. D. , Hanssen, F. , Muñoz, A.‐R. , Onrubia, A. , Wikelski, M. , … Silva, J. P. (2020). Wind turbines cause functional habitat loss for migratory soaring birds. Journal of Animal Ecology, 89(1), 93–103. 10.1111/1365-2656.12961 30762229

[ece36701-bib-0058] Masden, E. A. , Haydon, D. T. , Fox, A. D. , Furness, R. W. , Bullman, R. , & Desholm, M. (2009). Barriers to movement: Impacts of wind farms on migrating birds. ICES Journal of Marine Science, 66(4), 746–753. 10.1093/icesjms/fsp031

[ece36701-bib-0059] McDuie, F. , Casazza, M. L. , Overton, C. T. , Herzog, M. P. , Hartman, C. A. , Peterson, S. H. , … Ackerman, J. T. (2019). GPS tracking data reveals daily spatio‐temporal movement patterns of waterfowl. Movement Ecology, 7(1), 1–17. 10.1186/s40462-019-0146-8 30834128 PMC6388499

[ece36701-bib-0060] Meattey, D. E. , McWilliams, S. R. , Paton, P. W. C. , Lepage, C. , Gilliland, S. G. , Savoy, L. , … Osenkowski, J. E. (2019). Resource selection and wintering phenology of White‐winged Scoters in southern New England: Implications for offshore wind energy development. Condor, 121, 1–18. 10.1093/condor/duy014

[ece36701-bib-0061] Mo, Y. , Xie, H. , Li, B. , Zhang, W. , Tang, C. , & Wang, T. (2017). Community characteristics and habitat analysis of wintering Waterfowls between Different Manage Patterns of Rice paddy in Chongming Dongtan. Chinese Journal of Zoology, 52(4), 583–591. 10.13859/j.cjz.201704005

[ece36701-bib-0062] National Development and Reform Commission of the People’s Republic of China . (2016). The 13th five‐year plan for renewable energy development. Retrieved from http://www.ndrc.gov.cn/fzgggz/fzgh/ghwb/gjjgh/201706/W020170614416770246673.pdf

[ece36701-bib-0063] Niu, J. Y. , Zou, Y. A. , Yuan, X. , Zhang, B. , & Wang, T. H. (2013). Waterbird distribution patterns and environmentally impacted factors in reclaimed coastal wetlands of the eastern end of Nanhui county, Shanghai, China. Acta Zoologica Academiae Scientiarum Hungaricae, 59(2), 171–185.

[ece36701-bib-0064] Palumbo, M. D. , Petrie, S. A. , Schummer, M. , Rubin, B. D. , & Bonner, S. (2019). Mallard resource selection trade‐offs in a heterogeneous environment during autumn and winter. Ecology and Evolution, 9(4), 1798–1808. 10.1002/ece3.4864 30847073 PMC6392399

[ece36701-bib-0065] Papers, R. , Pomeroy, A. C. , Seaman, D. A. A. , Butler, R. W. , Elner, R. W. , Williams, T. D. , & Ydenberg, R. C. (2008). Feeding–danger trade‐offs underlie stopover site selection by migrants. Wildlife Research, 3(1), 7.

[ece36701-bib-0066] Parejo, M. , Gutiérrez, J. S. , Navedo, J. G. , Soriano‐Redondo, A. , Abad‐Gómez, J. M. , Villegas, A. , … Masero, J. A. (2019). Day and night use of habitats by Northern Pintails during winter in a primary rice‐growing region of Iberia. PLoS One, 14(7), e0220400 10.1371/journal.pone.0220400 31344107 PMC6658120

[ece36701-bib-0067] Pearse, A. T. , Brandt, D. A. , & Krapu, G. L. (2016). Wintering Sandhill Crane exposure to wind energy development in the central and southern Great Plains, USA. Condor, 118(2), 391–401. 10.1650/condor-15-99.1

[ece36701-bib-0068] Peters, K. A. , & Otis, D. L. (2007). Shorebird roost‐site selection at two temporal scales: Is human disturbance a factor? Journal of Applied Ecology, 44(1), 196–209. 10.1111/j.1365-2664.2006.01248.x

[ece36701-bib-0069] Plonczkier, P. , & Simms, I. C. (2012). Radar monitoring of migrating Pink‐footed Geese: Behavioural responses to offshore wind farm development. Journal of Applied Ecology, 49(5), 1187–1194. 10.1111/j.1365-2664.2012.02181.x

[ece36701-bib-0070] R Core Team . (2019). R: A Language and Environment for Statistical Computing. Vienna, Austria: R Foundation for Statistical Computing Retrieved from https://www.r‐project.org

[ece36701-bib-0071] Reid, T. , Krüger, S. , Whitfield, D. P. , & Amar, A. (2015). Using spatial analyses of Bearded Vulture movements in southern Africa to inform wind turbine placement. Journal of Applied Ecology, 52(4), 881–892. 10.1111/1365-2664.12468

[ece36701-bib-0072] Shaffer, J. A. , & Buhl, D. A. (2016). Effects of wind‐energy facilities on breeding grassland bird distributions. Conservation Biology, 30(1), 59–71. 10.1111/cobi.12569 26213098

[ece36701-bib-0073] State Forestry and Grassland Administration of China (2019). State Forestry and Grassland Administration of China on regulating the use of forest land for the construction of wind farm projects. Retrieved from http://www.forestry.gov.cn/main/4818/20190311/161520767440955.html

[ece36701-bib-0074] Tang, W. , Li, X. , Hu, C. H. , Zhu, C. , Li, Z. , Wu, D. , … He, G. (2019). Isolation of H8N4 avian influenza virus from wild birds in Shanghai. China. Acta Virologica, 63(1), 121–125. 10.4149/av_2019_116 30879322

[ece36701-bib-0075] Thaker, M. , Zambre, A. , & Bhosale, H. (2018). Wind farms have cascading impacts on ecosystems across trophic levels. Nature Ecology and Evolution, 2(12), 1854–1858. 10.1038/s41559-018-0707-z 30397304

[ece36701-bib-0076] Thaxter, C. B. , Ross‐Smith, V. H. , Bouten, W. , Clark, N. A. , Conway, G. J. , Masden, E. A. , … Burton, N. H. K. (2019). Avian vulnerability to wind farm collision through the year: Insights from Lesser Black‐backed Gulls (*Larus fuscus*) tracked from multiple breeding colonies. Journal of Applied Ecology, 56(11), 2410–2422. 10.1111/1365-2664.13488

[ece36701-bib-0077] Tripp, E. A. , Lendemer, J. C. , & McCain, C. M. (2019). Habitat quality and disturbance drive lichen species richness in a temperate biodiversity hotspot. Oecologia, 190(2), 445–457. 10.1007/s00442-019-04413-0 31093760

[ece36701-bib-0078] Walker, J. , Rotella, J. J. , Schmidt, J. H. , Loesch, C. R. , Reynolds, R. E. , Lindberg, M. S. , … Stephens, S. E. (2013). Distribution of duck broods relative to habitat characteristics in the Prairie Pothole Region. Journal of Wildlife Management, 77(2), 392–404. 10.1002/jwmg.466

[ece36701-bib-0079] Wang, S. , Wang, S. , & Smith, P. (2015). Ecological impacts of wind farms on birds: Questions, hypotheses, and research needs. Renewable and Sustainable Energy Reviews, 44, 599–607. 10.1016/j.rser.2015.01.031

[ece36701-bib-0080] Winder, V. L. , Gregory, A. J. , McNew, L. B. , & Sandercock, B. K. (2015). Responses of male Greater Prairie‐Chickens to wind energy development. Condor, 117(2), 284–296. 10.1650/CONDOR-14-98.1

[ece36701-bib-0081] Xia, S. , Yu, X. , Millington, S. , Liu, Y. U. , Jia, Y. , Wang, L. , … Jiang, L. (2017). Identifying priority sites and gaps for the conservation of migratory waterbirds in China’s coastal wetlands. Biological Conservation, 210, 72–82. 10.1016/j.biocon.2016.07.025

[ece36701-bib-0082] Xie, H. , Zhang, W. , Li, B. , Ma, Q. , & Wang, T. (2019). Industrial rice farming supports fewer waterbirds than traditional farming on Chongming Island, China. Ecological Research, 34(2), 286–295. 10.1111/1440-1703.1056

[ece36701-bib-0088] Yang, X. , Niu, J. , Luo, Z. , Zhang, M. , Tang, C. , & Wang, T. (2013). The impact of natural succession process on waterbird community in a abandoned fishpond at Chongming Dongtan, China. Acta Ecologica Sinica, 33(13), 4050–4058. 10.5846/stxb201207110978

[ece36701-bib-0083] Yetter, A. P. , Hagy, H. M. , Horath, M. M. , Lancaster, J. D. , Hine, C. S. , Smith, R. V. , & Stafford, J. D. (2018). Mallard survival, movements, and habitat use during autumn in Illinois. Journal of Wildlife Management, 82(1), 182–191. 10.1002/jwmg.21346

[ece36701-bib-0084] Zhang, P. , Zou, Y.‐A. , Xie, Y. , Zhang, S. , Chen, X. , Li, F. , … Tu, W. (2020). Hydrology‐driven responses of herbivorous geese in relation to changes in food quantity and quality. Ecology and Evolution, 10(12), 1–12. 10.1002/ece3.6272 PMC7319142 32607151

[ece36701-bib-0085] Zhang, W. , Li, X. , Yu, L. , & Si, Y. (2018). Multi‐scale habitat selection by two declining East Asian waterfowl species at their core spring stopover area. Ecological Indicators, 87, 127–135. 10.1016/j.ecolind.2017.12.035

[ece36701-bib-0086] Zou, Y. A. , Tang, C. D. , Niu, J. Y. , Wang, T. H. , Xie, Y. H. , & Guo, H. (2016). Migratory waterbirds response to coastal habitat changes: Conservation implications from Long‐term detection in the Chongming Dongtan wetlands, China. Estuaries and Coasts, 39(1), 273–286. 10.1007/s12237-015-9991-x

[ece36701-bib-0087] Zou, Y.‐A. , Zhang, P.‐Y. , Zhang, S.‐Q. , Chen, X.‐S. , Li, F. , Deng, Z.‐M. , … Xie, Y.‐H. (2019). Crucial sites and environmental variables for wintering migratory waterbird population distributions in the natural wetlands in East Dongting Lake, China. Science of the Total Environment, 655, 147–157. 10.1016/j.scitotenv.2018.11.185 30469060

