# Supplementary material for: Effect of wind farms on wintering ducks at an important wintering ground in China along the East Asian–Australasian Flyway
Source: Ecol Evol. 2020 Aug 20;10(17):9567–80. doi: 10.1002/ece3.6701 (PMC7487223; doi:10.1002/ece3.6701)
Supplement: Supplementary file 1 — Table S1‐S5 [file ECE3-10-9567-s001.docx]

### Table S1

| Habitat type | Survey area (ha) | | Total area |
| --- | --- | --- | --- |
|  | IWF | OWF |  |
| ABM | 533.65 | 546.34 | 1079.99 |
| PF | 129.08 | 131.19 | 260.27 |
| AP | 25.05 | 25.96 | 51.01 |
| ABM = artificial brackish marsh, PF = paddy field, AP = aquaculture pond, IWF = inside wind farm sites, OWF = outside wind farm sites. | | | |

### Table S2

| ID | Species | Age | Sex | Tarsus  length | Wing length | Bill  length | Body  mass | Transmitter  mass |
| --- | --- | --- | --- | --- | --- | --- | --- | --- |
|  |  |  | ♂/♀ | (mm) | (mm) | (mm) | (g) | (g) |
| **HSC023** | **Eastern Spot-billed duck** | **Adult** | **♂** | **47.01** | **28.20** | **65.73** | **1125.40** | **17.00** |
| **HSC024** | **Eastern Spot-billed duck** | **Adult** | **♂** | **42.82** | **26.20** | **61.45** | **1125.30** | **17.00** |
| **HSC025** | **Eastern Spot-billed duck** | **Adult** | **♂** | **43.95** | **28.30** | **61.18** | **1125.10** | **17.00** |
| **HSC026** | **Eastern Spot-billed duck** | **Adult** | **♀** | **44.19** | **26.80** | **59.05** | **1112.00** | **17.00** |
| **HSC027** | **Eastern Spot-billed duck** | **Adult** | **♀** | **38.25** | **26.60** | **60.93** | **1070.20** | **17.00** |
| **HSC028** | **Eastern Spot-billed duck** | **Adult** | **♀** | **37.83** | **24.80** | **54.15** | **1002.10** | **17.00** |
| Mean | (Eastern spot-billed duck) | | | 42.34 | 26.82 | 60.42 | 1093.35 |  |
| SD |  |  |  | 3.61 | 1.31 | 3.78 | 49.53 |  |
| **HSC021** | **Mallard** | **Adult** | **♀** | **44.10** | **27.00** | **56.72** | **1020.10** | **17.00** |
| **HSC029** | **Mallard** | **Adult** | **♀** | **36.75** | **26.50** | **60.51** | **955.10** | **17.00** |
| **HSC030** | **Mallard** | **Adult** | **♀** | **38.62** | **22.50** | **52.61** | **835.10** | **14.00** |
| HSC031 | Mallard | Adult | ♂ | 35.76 | 20.6 | 60.6 | 890.10 | 14.00 |
| HSC032 | Mallard | Adult | ♀ | 40.51 | 21.0 | 61.19 | 865.30 | 14.00 |
| HSC046 | Mallard | Adult | ♂ | 35.2 | 21.3 | 58.89 | 835.60 | 14.00 |
| Mean | (Mallard) | | | 38.49 | 23.15 | 58.42 | 900.22 |  |
| SD |  |  |  | 3.37 | 2.86 | 3.28 | 73.65 |  |

SD = standard deviation.

### Table S3

| Variable | Description |
| --- | --- |
| Distance to wind turbine (DWT) | Distance to the nearest wind turbine (km). |
| Distance to water (DW) | Distance to edge of mudflat, natural brackish marsh, artificial brackish marsh, aquaculture pond, ditch or canal covered with water (m). |
| Index of human activity (IHA) | Summary of 3 metrics related to duck daily human activity per unit including: number of motorized vehicles passing through (0–3), distance to road (0-3), and various construction activities duration/day (0-3) (Table S4). |
| Land cover (LC) | Type of land use in study area: natural brackish marsh, artificial brackish marsh, paddy field, and others (mudflat, upland field, ditch, canal) according to artificial visual interpretation and field survey. |

### Table S4

| Category | Level | Score |
| --- | --- | --- |
| Maximum number of motorized vehicles passing through | Level 1: ( motorized vehicles = 0) | 0 |
|  | Level 2: ( 0 < motorized vehicles ≤5) | 1 |
|  | Level 3: ( 5 < motorized vehicles ≤ 10) | 2 |
|  | Level 4: ( motorized vehicles > 10) | 3 |
| Distance to road (m) | Level 1: ( distance to road = 0) | 3 |
|  | Level 2: ( 0 < distance to road ≤ 300 m) | 2 |
|  | Level 3: ( 300 < distance to road ≤ 1000 m) | 1 |
|  | Level 4: ( distance to road > 1000 m) | 0 |
| Construction activity (h) | Level 1: ( duration time = 0) | 0 |
|  | Level 2: ( 0 < duration time ≤ 1 h) | 1 |
|  | Level 3: ( 1 < duration time ≤ 3 h) | 2 |
|  | Level 4: ( duration time > 3 h) | 3 |

### Table S5

| Period | Model parameters | *k* | ΔAIC_C_ | Log(L) | w*_i_* |
| --- | --- | --- | --- | --- | --- |
| Day | DW + DWT + IHA + LC | 9 | 0.00 | −61.72 | 0.81 |
|  | DW + DWT + IHA | 6 | 3.16 | −66.46 | 0.17 |
|  | DW + DWT + LC | 8 | 7.62 | −66.59 | 0.02 |
|  | DW + IHA + LC | 8 | 9.58 | −67.57 | 0.01 |
|  | DWT + LC | 7 | 26.55 | −77.11 | 0.00 |
|  | DWT+IHA | 5 | 26.56 | −79.21 | 0.00 |
|  | DWT + IHA + LC | 8 | 29.02 | −77.29 | 0.00 |
|  | IHA + LC | 7 | 31.44 | −79.56 | 0.00 |
|  | DW + DWT | 5 | 38.95 | −85.40 | 0.00 |
|  | LC | 6 | 45.75 | −87.76 | 0.00 |
|  | DW+LC | 7 | 47.80 | −87.74 | 0.00 |
|  | DWT | 4 | 55.93 | -94.93 | 0.00 |
|  | IHA | 4 | 60.10 | −97.01 | 0.00 |
|  | DW+IHA | 5 | 62.06 | −96.96 | 0.00 |
|  | DW | 4 | 105.59 | −119.76 | 0.00 |
|  | Null | 3 | 130.94 | −133.46 | 0.00 |
| Night | DWT+DW+LC | 8 | 0.00 | −205.05 | 0.65 |
|  | DWT+DW+IHA+LC | 9 | 1.22 | −204.62 | 0.35 |
|  | DW+LC+IHA | 8 | 22.16 | −216.13 | 0.00 |
|  | DW+LC | 7 | 22.60 | −217.39 | 0.00 |
|  | DWT+LC | 7 | 60.41 | −239.29 | 0.00 |
|  | DWT+LC+IHA | 8 | 61.79 | −235.95 | 0.00 |
|  | DWT+DW+IHA | 6 | 69.08 | −241.66 | 0.00 |
|  | DWT+DW | 5 | 77.30 | −246.80 | 0.00 |
|  | DW+IHA | 5 | 77.61 | −246.95 | 0.00 |
|  | DW | 4 | 87.77 | −253.06 | 0.00 |
|  | LC | 6 | 95.52 | −254.88 | 0.00 |
|  | IHA+LC | 7 | 97.54 | −254.86 | 0.00 |
|  | DWT | 4 | 141.61 | −279.98 | 0.00 |
|  | DWT+IHA | 5 | 143.56 | −279.93 | 0.00 |
|  | NULL | 3 | 199.09 | −309.73 | 0.00 |
|  | IHA | 4 | 199.56 | −308.95 | 0.00 |

DWT = distance to wind turbine, DW = distance to water, IHA = index of human activity, LC = land cover.
